# Overexpression of CTNND1 in hepatocellular carcinoma promotes carcinous characters through activation of Wnt/β-catenin signaling

**DOI:** 10.1186/s13046-016-0344-9

**Published:** 2016-05-18

**Authors:** Bo Tang, Fang Tang, Zhenran Wang, Guangying Qi, Xingsi Liang, Bo Li, Shengguang Yuan, Jie Liu, Shuiping Yu, Songqing He

**Affiliations:** Department of Hepatobiliary Surgery, Guilin Medical University, Affiliated Hospital, Guilin, 541001 Guangxi People’s Republic of China; Laboratory of Liver Injury and Repair Molecular Medicine, Guilin Medical University, Guilin, 541001 Guangxi People’s Republic of China; Department of Pathology and Physiopathology, Guilin Medical University, Guilin, 541004 Guangxi People’s Republic of China

**Keywords:** CTNND1, Hepatocellular carcinoma, Migration, Metastasis

## Abstract

**Background:**

Increasing evidence supports the association of CTNND1 with tumor development and progression. However, the mechanism and clinical significance of CTNND1 deregulation in hepatocellular carcinoma (HCC) remains unknown. In this study, we aim to investigate the role of CTNND1 in HCC.

**Methods:**

qRT-PCR and immunohistochemical analyses were used to measure the levels of CTNND1 in HCC specimens and HCC cell lines. CTNND1 and shCTNND1 were transfected into HCC cell lines to investigate its role in HCC. Cell migration and invasion were measured by Transwell and Matrigel analyses in vitro. In vivo metastasis assays were performed in SCID mice.

**Results:**

In clinical HCC samples, we found that CTNND1 expression was significantly up-regulated in cancer lesions compared with paired normal liver tissues. By silencing or overexpressing CTNND1 in HCC cells, we found that CTNND1 could promote cell proliferation, migration, and invasion in vitro. An in-vivo assay showed that CTNND1 dramatically promoted HCC cell tumor formation and metastasis. Moreover, CTNND1 promoted HCC metastasis, at least in part, by indirectly enhancing Wnt/β-catenin signaling. Consistent with these results, the expression of CTNND1 was positively correlated with β-catenin, WNT11, Cyclin D1, and BMP7 expression in human HCC specimens.

**Conclusions:**

Our study provides evidence that CTNND1 functions as a novel tumor oncogene in HCC, and may be a potential therapeutic target for HCC management.

**Electronic supplementary material:**

The online version of this article (doi:10.1186/s13046-016-0344-9) contains supplementary material, which is available to authorized users.

## Background

Hepatocellular carcinoma (HCC) is the third leading cause of cancer deaths worldwide [1–3]. The vast majority of HCCs arise in the context of chronic liver injury, inflammation, and hepatocyte proliferation provoked by different etiologies [4, 5]. HCCs have a highly variable clinical course, and include several subgroups with distinct pathways of hepatocarcinogenesis [6]. These processes share common mechanisms with embryogenesis and can be considered as an aberrant form of organogenesis [7, 8]. Surgical resection and liver transplantation are available options for the treatment of early-stage HCC; however, the prognosis of HCC remains poor because of a high level of tumor invasiveness, frequent intrahepatic spread, extrahepatic metastasis, and resistance to chemotherapy.

Cell - cell contact is initiated and maintained by the adherens junction (AJ). AJs are complexes that are located at the basolateral membrane, and are composed of classical cadherins [9]. The cadherin cytoplasmic domain directly interacts with CTNND1 (δ-catenin) and β-catenin (CTNNB1). Together with α-catenin (CTNNA1), which binds to β-catenin and links the AJ to the actin cytoskeleton, they form the core AJ complex [10]. Accumulating evidence has revealed that the Armadillo catenins are multifunctional nucleo-cytoplasmic shuttling proteins that regulate gene expression via interaction with DNA-binding transcriptional factors [10]. This activity is best illustrated by β-catenin, which is translocated into the nucleus, where it functions as a co-transcriptional factor in Wnt/β-catenin signaling. In contrast to β-catenin, the role of CTNND1 has been less elucidated. CTNND1 first attracted attention when the known transcriptional factor Kaiso was identified as a CTNND1-specific binding partner [11]. Kaiso inhibits β-catenin/TCF activation of target genes, and CTNND1 inhibits Kaiso activity. Thus, it is now thought that the Kaiso/CTNND1 complex is a modulator of the canonical Wnt/β-catenin signaling pathway [11]. Recently, a large amount of data has implicated CTNND1 in the regulation of cancer development and progression [12–14]. CTNND1 can function as an oncogene that drives migration and anchorage independence, both of which are established hallmarks of metastatic cancer [15, 16]. These results suggest that CTNND1 plays a role in the oncogenesis of some malignancies. However, whether CTNND1 expression contributes to HCC development and progression is not known.

In the present study, we sought to determine whether and, if so, how CTNND1 regulates the growth and invasion of HCC cells. We found that CTNND1 expression was significantly up-regulated in cancer lesions compared with paired normal liver tissues. By silencing or overexpressing CTNND1 in HCC cells, we found that CTNND1 could promote cell proliferation, migration, and invasion in vitro. An in-vivo assay showed that CTNND1 dramatically promoted HCC cell tumor formation and metastasis. Moreover, CTNND1 promoted HCC metastasis, at least in part, by indirectly interacting with Wnt/β-catenin to enhance Wnt/β-catenin signaling. Consistent with these results, the expression of CTNND1 was positively correlated with β-catenin, WNT11, Cyclin D1, and MMP7 expression in human HCC specimens. Our study provides evidence that CTNND1 functions as a novel tumor oncogene in HCC, and may be a potential therapeutic target for HCC management.

## Methods

### Chemicals and antibodies

Lipofectamine 2000 transfection and Trizol LS reagents were purchased from Invitrogen (Grand Island, NY, USA). Antibodies against CTNND1, WNT11, β-catenin, Cyclin D1, MMP7, and Kaiso were purchased from Abcam (Cambridge, MA, USA). E-cadherin, N-cadherin, vimentin, and β-actin antibodies were from Cell Signaling Technology (Danvers, MA, USA). Anti-α-catenin antibody was from BD (Franklin Lakes, NJ, USA).

### Patients and specimens

Twenty-six tumor and para-cancerous tissues, which were used for qRT-PCR analysis, were randomly collected from HCC patients who underwent curative resection with informed consent between 2013 and 2014 at the Department of Laparoscopic Surgery, First Affiliated Hospital of Guilin Medical University. All tissues were collected immediately upon resection of the tumors in the operating theater, transported in liquid nitrogen, and then stored at -80 °C. Another 289 hepatocellular carcinoma tissues, which were used for immunohistochemical analysis, were randomly collected from HCC patients who underwent curative resection with informed consent between 2007 and 2014 at the Department of Hepatobiliary Surgery, First Affiliated Hospital of Guilin Medical University. Tumor staging was based on the 6th edition of the tumor-node-metastasis (TNM) classification of the International Union Against Cancer. The clinicopathologic characteristics of the 289 hepatocellular carcinoma tissues are summarized in Table 1. Follow-up data were summarized at the end of March 2015, with a median observation time of 66.8 months. Study protocols were approved by the Hospital Ethics Committee of Guilin Medical University, and written informed consent was obtained from patients based on the Declaration of Helsinki.

**Table 1:** CTNND1 staining and clinicopathologic characteristics of 289 hepatocellular carcinoma patients

Variables	CTNND1 staining		Total	*P* ^a^
	Low	High		
Age (y)				0.667
≤ 50	89 (77 %)	27 (23 %)	116	
> 50	131 (76 %)	42 (24 %)	173	
Sex				0.262
Male	163 (69 %)	27 (31 %)	235	
Female	42 (78 %)	12 (22 %)	54	
HBsAg				0.231
Negative	53 (65 %)	28 (35 %)	81	
Positive	151 (73 %)	57 (27 %)	208	
HCV				0.135
Negative	185 (76 %)	57 (24 %)	242	
Positive	31 (66 %)	16 (34 %)	47	
AFP				0.634
≤ 20	64 (69 %)	27 (31 %)	93	
> 20	136 (68 %)	60 (32 %)	196	
γ-GT(U/L)				0.187
≤ 54	113 (81 %)	26 (19 %)	139	
> 54	110 (73 %)	40 (27 %)	150	
Liver cirrhosis				0.766
No	41 (66 %)	22 (34 %)	62	
Yes	159 (70 %)	68 (30 %)	227	
Tumor diameter (cm)				0.028
≤ 5	178 (70 %)	77 (30 %)	255	
> 5	15 (44 %)	19 (56 %)	34	
Microvascular invasion				<0.001
Absence	92 (80 %)	23 (20 %)	115	
Present	63 (36 %)	111 (64 %)	174	
Tumor encapsulation				0.196
Complete	83 (62 %)	50 (38 %)	133	
None	91 (58 %)	65 (42 %)	156	
Tumor differentiation				<0.001
I + II	135 (71 %)	56 (29 %)	191	
III + IV	31 (32 %)	67 (68 %)	98	
TNM stage				0.307
I	161 (69 %)	73 (31 %)	234	
II + III	32 (58 %)	23 (42 %)	55	

*Abbreviations: HBsAg* hepatitis B surface antigen; *AFP* α-fetoprotein; *γ-GT* γ-glutamyl transferase; TNM, tumor-nodesmetastasis

^a^ *p*-value < 0.05 was considered statistically significant. *p*-values were calculated using the Pearson chi-square test

### Histological and immunohistochemical analysis

The normal human liver tissues, human tumor tissues, and lungs dissected from mice were fixed in 4 % paraformaldehyde in phosphate-buffered saline (PBS) overnight and subsequently embedded in paraffin wax. Sections cut at a thickness of 4 μm were stained with hematoxylin and eosin for histological analysis. Immunohistochemical analysis was performed for different markers in these arrays as described previously. The proportion of stained cells (low, <30 % staining; high, ≥30 % staining) was semiquantitatively determined following published protocols [17, 18].

### Cell culture

HCC cells (ATCC, Manassas, VA, USA) were cultured under the following conditions: SNU-449, HCCLM3, HepG2, SK-Hep-1, and MHCC97H cell lines were cultured using 10 % fetal bovine serum (Invitrogen) in RPMI-1640 (Invitrogen). The HL7702 cell line was cultured using 10 % fetal bovine serum in Dulbecco's modified Eagle medium (Invitrogen). Cell culture was according to the manufacturer’s protocol. All the cell lines were grown at 37 °C in a 5 % CO_2_/95 % air atmosphere, and were revived every 3 to 4 months.

### Establishment of CTNND1 stable expression and knockdown cell lines

Retroviral constructs containing human pBabe-*CTNND1* cDNA and pSuper.retro.puro with shRNA against human *CTNND1* were prepared as described previously [17, 19]. The generation of retrovirus supernatants and transfection of hepatocellular carcinoma cells were conducted as described previously [17, 20]. The expression of *CTNND1* was confirmed by qRT-PCR and Western blotting analysis.

### MTT assay

The transfected cells were seeded in 96-well plates at a density of 3× 103 cells/well. MTT solution (20 μ of 5 mg/ml MTT) was added to each well (for a total volume of 250 μl), and the plates were incubated for 4 h at 37 °C. Following the removal of the culture medium, the remaining crystals were dissolved in DMSO, and the absorbance was measured at 570 nm using a microplate reader. Cell proliferation was assessed daily for four consecutive days.

### Wound-healing assay

Cells were seeded in 6-cm culture plates. Cell monolayers were wounded by scratching with sterile plastic 200-μl micropipette tips, and were photographed using phase-contrast microscopy. The migration distance of each cell was measured after the photographs were converted to Photoshop files.

### Cell invasion and motility assays

Invasion of cells was measured in Matrigel-coated (BD, Franklin Lakes, NJ, USA) Transwell inserts (6.5 mm, Costar, Manassas, VA, USA) containing polycarbonate filters with 8-μm pores as detailed previously [21, 22]. The inserts were coated with 50 μl of 1 mg/ml Matrigel matrix according to the manufacturer’s recommendations. Cells (2 × 10^5^) in 200 μl of serum-free medium were plated in the upper chamber, and 600 μl of medium with 10% fetal bovine serum was added to lower well. After a 24-h incubation, cells that had migrated to the lower surface of the membrane were fixed and stained. For each membrane, five random fields were counted at × 10 magnification. Motility assays were similar to Matrigel invasion assays except that the Transwell insert was not coated with Matrigel.

### Western blotting

Cells were lysed in lysis buffer and total protein contents were determined by the Bradford method. Proteins (30 μg) were separated by reducing SDS-PAGE, and probed with specific antibodies. Blots were washed and probed with respective secondary peroxidase-conjugated antibodies, and the bands were visualized by chemoluminescence (Amersham Biosciences, Shanghai, China).

### Confocal immunofluorescence microscopy

Cell lines were plated onto culture slides (Costar, Manassas, VA, USA). After 24 h, the cells were rinsed with PBS and fixed with 4 % paraformaldehyde in PBS. Cell membranes were permeabilized using 0.5 % Triton X-100. The cells were then blocked for 30 min in 10 % BSA in PBS, and then incubated with primary antibodies in 10 % BSA overnight at 4 °C. After three washes in PBS, the slides were incubated for 1 h in the dark with FITC-conjugated secondary goat anti-mouse, or goat anti-rabbit antibodies (Invitrogen). After three further washes, the slides were stained with DAPI for 5 min to visualize the nuclei, and were examined using a Carl Zeiss confocal imaging system (LSM 780; Carl Zeiss, Jena, Germany).

### qRT-PCR

Total RNA was extracted using Trizol reagent, and cDNA was synthesized using SuperScript-Reverse Transcriptase (Invitrogen). qRT-PCR and data collection were performed with an ABI PRISM 7900HT sequence detection system. The primers used for the amplification of the indicated genes are available upon request.

### Gene expression profiling

Total RNA quality and quantity were determined using an Agilent 2100 Bioanalyzer and NanoDrop ND-1000. Affymetrix HU U133 plus 2.0 arrays were used according to the manufacturer’s protocol. The data were initially normalized by robust multiarray average (RMA) normalization algorithms in the expression console software (Affymetrix). Significantly altered genes between CTNND1-overexpressing cells and the control cells were considered by scatter plots and the genes up- and down-regulated by ≥5-fold. Clustering analysis was done using a gene list by Gene Cluster v3.0 software, and heat maps were visualized using Java TreeView v1.1.4r3 software. Gene-set enrichment analysis was carried out using ConceptGen. Gene sets were either obtained from ConceptGen or from published gene signatures.

### In-vivo tumor growth and metastasis

Nude mice were purchased from the Shanghai Slac Laboratory Animal Co. Ltd., and were maintained in microisolator cages. All animals were used in accordance with institutional guidelines, and the current experiments were approved by the Use Committee for Animal Care. For subcutaneous inoculation, different numbers of tumor cells were resuspended in PBS medium with 50 % Matrigel, and were inoculated subcutaneously into the 8-week-old nude mice. The tumors were measured weekly, and the tumor volume was calculated according to the formula: length × width2/2. The mice were killed 40 days after the inoculation. For metastasis assays, cells were resuspended in PBS at a concentration of 1x10^7^ cells ml^-1^. Cell suspension (0.1 ml; 1x10^6^ cells) was injected into tail veins of nude mice. All of the mice were killed by CO_2_ 60 days after inoculation.

### Statistical analysis

Results were analyzed with SPSS13.0 statistical software. Correlation between *CTNND1* expression and clinicopathologic parameters was evaluated using the Chi-square (*χ*2) test, and quantitative variables were analyzed by the independent *t* test. The survival probability was estimated by the Kaplan–Meier method, and the comparison of survival curves between groups was done with the log-rank test. The statistical significance of the differences between mean values was determined by *P* <0.05.

## Results

### *CTNND1* is highly expressed and correlated with distant metastasis in HCCs

**Fig. 1:**
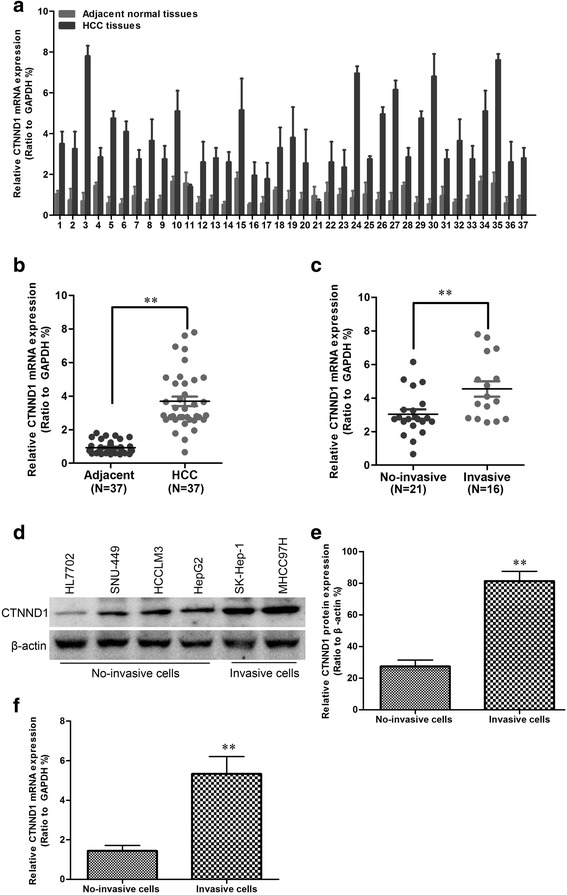
CTNND1 is highly expressed in HCC tissues and HCC cell lines. **a** *CTNND1* mRNA expression was analyzed by quantitative RT-PCR in tumors and adjacent tissues in 37 samples. **b** Comparison of the expression levels of *CTNND1* mRNA in adjacent normal tissues and HCC. **c** Comparison of the expression levels of *CTNND1* mRNA in non-invasive and invasive HCC tissues. **d** CTNND1 protein expression was analyzed by Western blotting in HL7702, SNU-449, HCCLM3, HepG2, SK-Hep-1, and MHCC97H cell lines. **e** Comparison of the expression levels of CTNND1 protein in non-invasive and invasive cell lines. **f** Comparison of the expression levels of *CTNND1* mRNA in non-invasive and invasive cell lines. **, *P* < 0.01 is based on the Student *t* test. All results are from three independent experiments. Error bars, SD

To investigate whether CTNND1 might be involved in HCC, the mRNA expression level of *CTNND1* in HCC tissues and its matched normal adjacent tissues was determined by qRT-PCR in 37 samples (Fig. 1a). Compared with normal tissues, HCC specimens showed overexpression of *CTNND1* (Fig. 1b). We then analyzed *CTNND1* expression in HCCs without or with invasive property; we found that *CTNND1* mRNA overexpression was significantly correlated with invasive property in HCC tissues (Fig. 1c). As shown in Additional file 1: Figure S1, the expression level of CTNND1 protein (Fig. 1d and e) and mRNA (Fig. 1f) in invasive HCC cell lines was higher than that in the non-invasive HCC cell lines. These data demonstrate that the upregulation of CTNND1 might be relevant to the invasive properties of HCC.

**Fig. 2:**
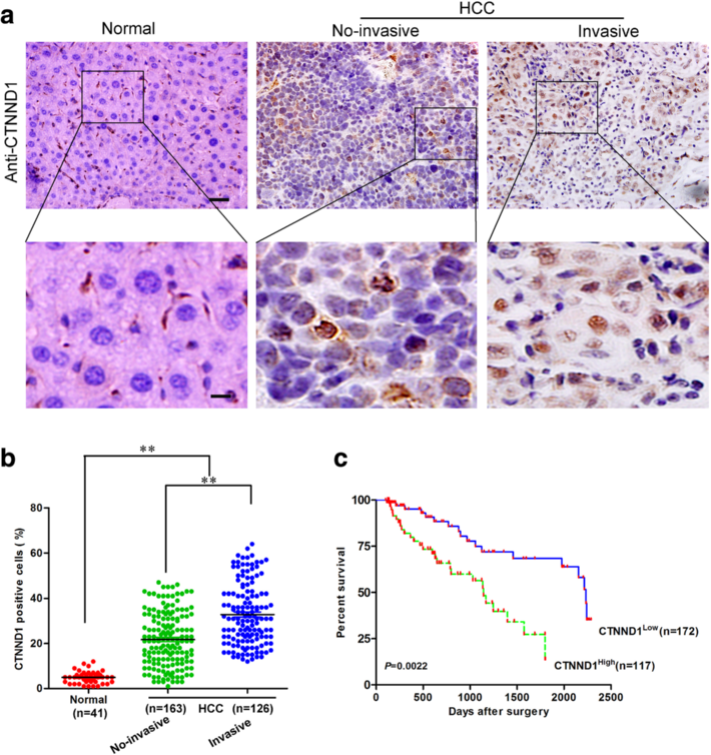
CTNND1 is correlated with distant metastasis in HCC tissues. **a** CTNND1 protein expression was analyzed by immunohistochemical analysis in 289 cases of HCC tissue, and representative results are shown. **b** Semiquantification of CTNND1 expression in normal tissues, and non-invasive and invasive HCC tissues. **c** The association between CTNND1 expression in HCC and the survival time of selected patients was analyzed by Kaplan–Meier survival analysis. Scale bars, 50 μm (*upper*) and 20 μm (*lower*) in C. **, *P* < 0.01 is based on the Student *t* test. All results are from three independent experiments. Error bars, SD

We examined CTNND1 protein expression in more HCC samples by IHC (Fig. 2a). We observed that the level of CTNND1-positive cells was markedly higher in HCC tissues than in the normal liver tissues (Fig. 2b). Most importantly, CTNND1 overexpression was consistently significantly correlated with distant metastasis in these HCC samples (Fig. 2b). To investigate the relationship between CTNND1 expression and clinicopathological parameters in the 289 cases with HCC, these cases were first divided into two subgroups: “low CTNND1 expression” and “high CTNND1 expression” as defined in the immunohistochemistry section of “Materials and methods”. Significant correlations were found between CTNND1 expression and tumor diameter, microvascular invasion, and tumor differentiation. There were no statistical correlations between CTNND1 expression and other clinicopathological parameters, such as patient age, gender, and HBsAg (Table 1). The association between CTNND1 expression in HCC and the survival time of selected patients was analyzed by Kaplan–Meier survival analysis (Fig. 2c). The median overall survival time of the high CTNND1 expression group was significantly shorter than that of the low CTNND1 expression group (*P* = 0.0022). In addition, we analyzed the expression of CTNND1 with TCGA data, and found that the overall survival time of the high CTNND1 expression group was also significantly shorter than that of the low CTNND1 expression group in TCGA data (Additional file 1: Figure S1). Together, these results indicate a functional role of CTNND1 in the aggressive behavior of HCC.

### CTNND1 promotes the proliferative capacity of HCC cells both in vitro and in vivo

**Fig. 3:**
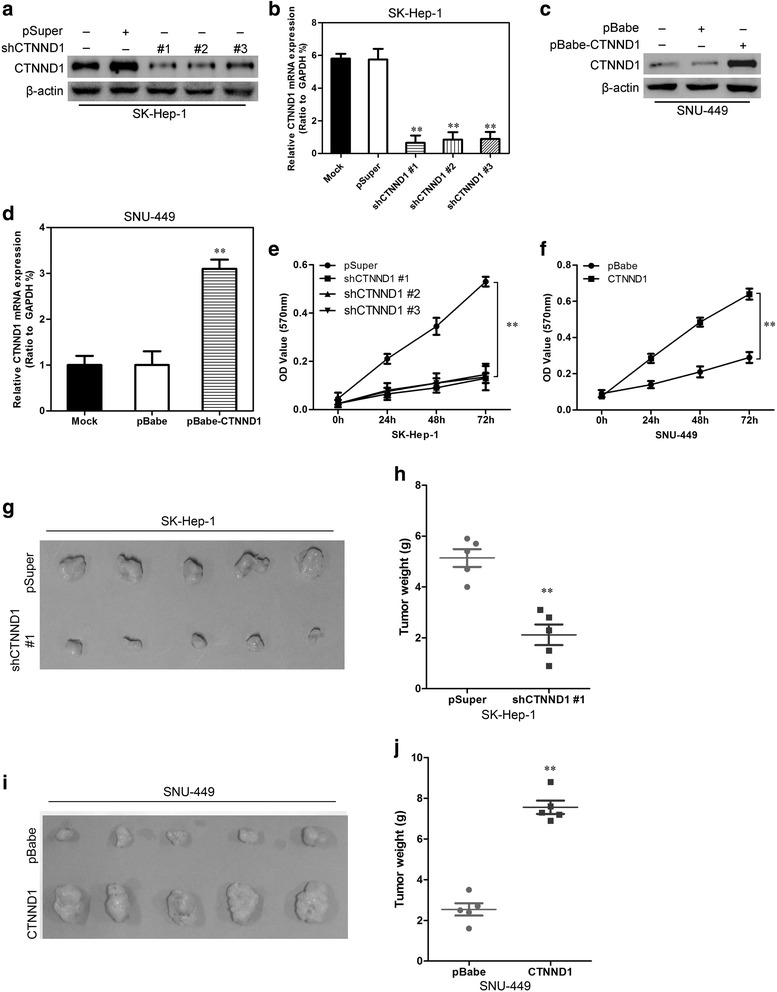
CTNND1 promotes proliferative capacity of HCC cells both in vitro and in vivo. **a** The expression of CTNND1 protein was measured by Western blotting in the SK-Hep-1-shCTNND1s cell line and theirs control cells. **b** The expression of *CTNND1* mRNA was measured by qRT-PCR in the SK-Hep-1-shCTNND1s cell line and theirs control cells. **c** The expression of CTNND1 protein was measured by Western blotting in the SNU-449-CTNND1 cell line and its control cells. **d** The expression of *CTNND1* mRNA was measured by qRT-PCR in the SNU-449-CTNND1 cell line and its control cells. **e** The proliferation of the SK-Hep-1-shCTNND1s cell line and theirs control cells was examined by MTT assay. **f** The proliferation of the SNU-449-CTNND1 cell line and its control cells was examined by MTT assay. **g** Representative images of SK-Hep-1-shCTNND1 #1 or its control cell tumors by subcutaneous injection. **h** The weight of tumors formed by SK-Hep-1-shCTNND1 #1 cells or its control cells at harvest. **i** Representative images of SNU-449-CTNND1 cell or control cell tumors by subcutaneous injection. **j** The weight of tumors formed by SNU-449-CTNND1 cells or its control cells at harvest. **, *P* < 0.01 is based on the Student *t* test. All results are from three independent experiments. Error bars, SD

In order to test the oncogenic activity of CTNND1 in HCC, we retrovirally established stable silencing of CTNND1 in SK-Hep-1 cells and overexpression of CTNND1 in SNU-449 cells. The levels of CTNND1 in these resultant cell lines were verified by Western blotting (Fig. 3a and c) and qRT-PCR (Fig. 3b and d).

Compared to vector-only controls, silencing of CTNND1 in SK-Hep-1 cells significantly reduced cell proliferation by the MTT assay (Fig. 3e), In contrast, overexpression of CTNND1 in SNU-449 cells significantly increased cell proliferation (Fig. 3f). To extend our in-vitro observations, we investigated whether CTNND1 could regulate tumorigenic capacity of HCC cells in vivo. SK-Hep-1-shCTNND1 #1 and SNU-449-CTNND1 cells, and their corresponding control cells, were subcutaneously injected into nude mice. As expected, silencing of CTNND1 in the typically aggressive SK-Hep-1 cells led to a dramatic decrease in tumor volume and weight (Fig. 3g and h). In contrast, SNU-449-CTNND1 cells grew more rapidly at the implantation site than the control cells (Fig. 3i and j). Taken together, these results suggest that CTNND1 is an important regulator of proliferation in HCC cells.

### CTNND1 promotes migratory and invasive capacities of HCC cells in vitro and metastasis in vivo

**Fig. 4:**
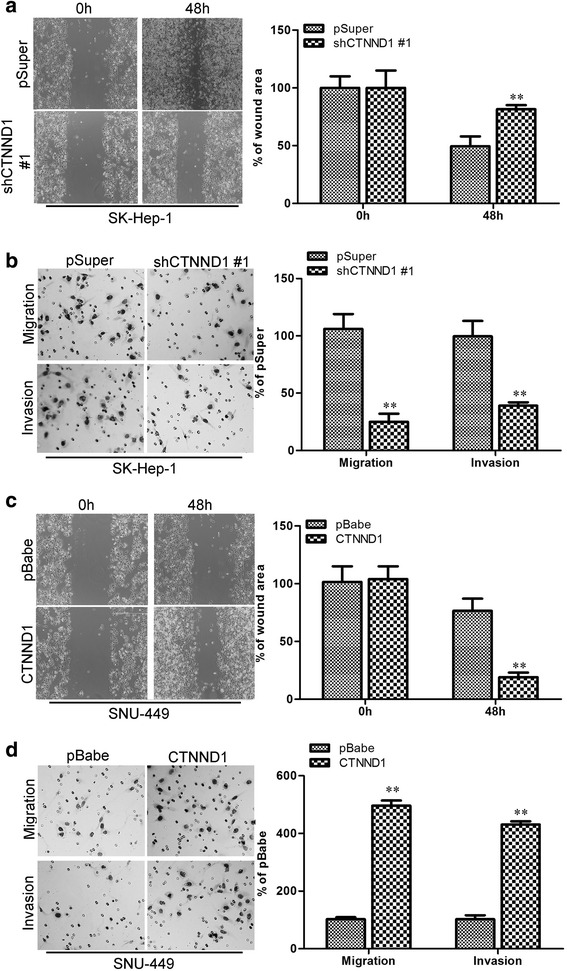
CTNND1 promotes migratory and invasive capacities of HCC cells in vitro. **a** The SK-Hep-1-shCTNND1 #1 cell line and its control cells were subjected to wound-healing assays; the uncovered areas in the wound-healing assays were quantified as a percentage of the original wound area. **b** The SK-Hep-1-shCTNND1 #1 cell line and its control cells were subjected to Transwell migration (*top*), and Matrigel invasion assays (*bottom*). Quantification of migrated cells through the membrane and invaded cells through Matrigel of each cell line are shown as proportions of their vector controls. **c** The SNU-449-CTNND1 cell line and its control cells were subjected to wound-healing assays. The uncovered areas in the wound-healing assays were quantified as a percentage of the original wound area. **d** The SNU-449-CTNND1 cell line and its control cells were subjected to Transwell migration (*top*) and Matrigel invasion assays (*bottom*). Quantification of migrated cells through the membrane and invaded cells through Matrigel of each cell line are shown as proportions of their vector controls. **, *P* < 0.01 is based on the Student *t* test. All results are from three independent experiments. Error bars, SD

The effect of CTNND1 on cell migration was first assessed using a wound-healing assay. Silencing CTNND1 dramatically reduced the migratory and invasive capacity of SK-Hep-1 cells (Fig. 4a and b). In contrast, SNU-449-CTNND1 cells had significantly faster closure of the wound area compared to their control cells (Fig. 4c). This result was confirmed using a Boyden's chamber assay (Fig. 4d). Moreover, Huh7-pBabe-CTNND1 and HepG2-pBabe-CTNND1 cells showed a greater degree of invasion through Matrigel (Fig. 4d). These results indicate that CTNND1 promotes migratory and invasive behavior in HCC cells.

**Fig. 5:**
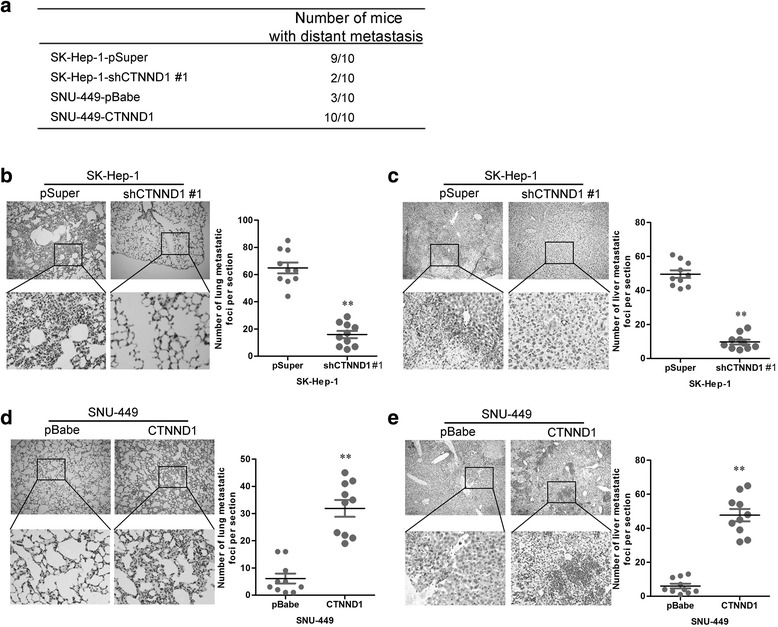
CTNND1 promotes HCC cell metastasis in vivo. **a** The total numbers of mice with distant metastasis at 60 days after injection of SK-Hep-1-shCTNND1 #1 and SNU-449-CTNND1 cells, or their control cells. **b** The numbers of metastatic foci per section in the lungs of individual mice with injection of SK-Hep-1-shCTNND1 #1 cells or control cells. **c** The numbers of metastatic foci per section in the liver of individual mice with injection of SK-Hep-1-shCTNND1 #1 cells or control cells. **d** The numbers of metastatic foci per section in the lungs of individual mice with injection of SNU-449-CTNND1 cells or control cells. **e** The numbers of metastatic foci per section in the liver of individual mice with injection of SNU-449-CTNND1 cells or control cells. *n* = 10 for tail vein injections. **, *P* < 0.01 is based on the Student *t* test. All results are from three independent experiments. Error bars, SD

We then investigated the functional relevance of CTNND1 to metastasis in vivo. SK-Hep-1-shCTNND1 #1 and SNU-449-CTNND1 cells, and their corresponding control cells, were injected into nude mice through the tail vein. CTNND1 overexpression not only significantly increased the number of mice with distant metastasis (Fig. 5a), but also dramatically increased the number of metastatic tumors in the lungs and liver of each mouse (Fig. 5d and e). Silencing CTNND1 in SK-Hep1 cells inhibited metastatic behavior, both in terms of the number of mice with distant metastasis (Fig. 5a) and the number of metastatic tumors in the lungs and liver of each mouse (Fig. 5b and c). Therefore, the in-vivo results further demonstrate the critical role of CTNND1 in HCC metastasis.

### CTNND1 regulates the transition between epithelial and mesenchymal phenotypes in HCC cells

**Fig. 6:**
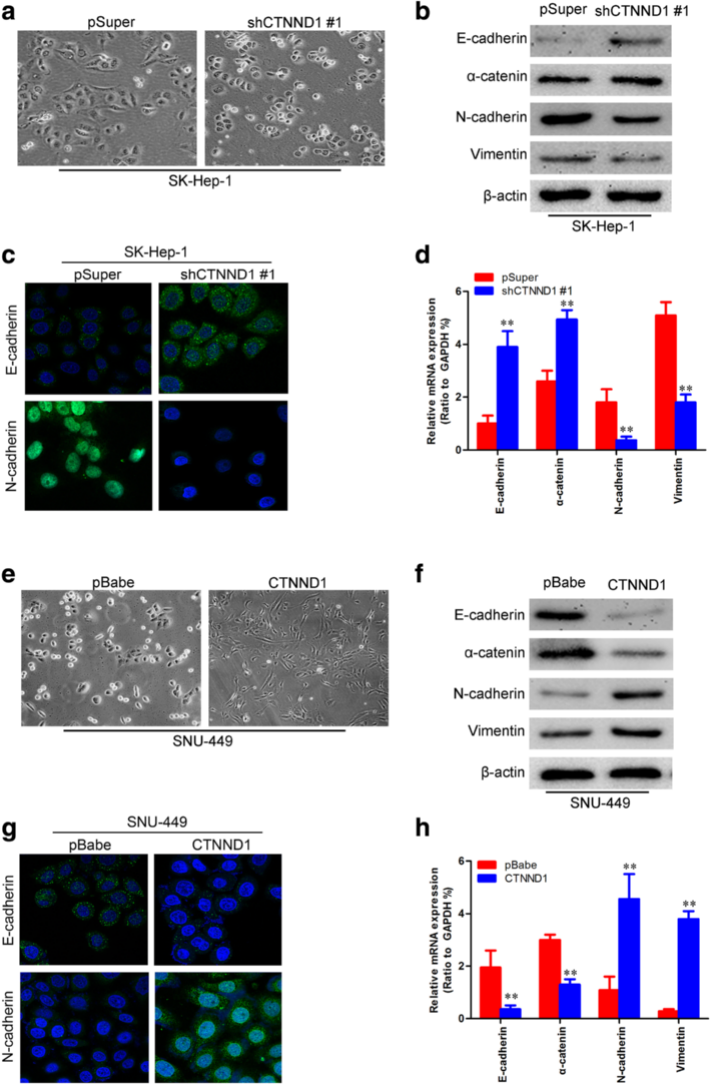
CTNND1 regulates the transition between epithelial and mesenchymal phenotypes in HCC cells. **a** Representative phase-contrast images of SK-Hep-1 cells showed that CTNND1 silencing modulated morphologic changes. **b** Expression of epithelial (E-cadherin and α-catenin) and mesenchymal (N-cadherin and Vimentin) markers was analyzed by Western blotting in SK-Hep-1-shCTNND1 #1 cells and control cells. **c** Expression of epithelial (E-cadherin) and mesenchymal (N-cadherin) markers was analyzed by immunofluorescence stains in SK-Hep-1-shCTNND1 #1 cells and control cells. **d** Expression of epithelial and mesenchymal markers was analyzed by qRT-PCR in SK-Hep-1-shCTNND1 #1 cells and control cells. **e** Representative phase-contrast images of SNU-449 cells showed that CTNND1 overexpression modulated morphologic changes. **f** Expression of epithelial (E-cadherin and α-catenin) and mesenchymal (N-cadherin and Vimentin) markers was analyzed by Western blotting in SNU-449-CTNND1 cells and control cells. **g** Expression of epithelial (E-cadherin) and mesenchymal (N-cadherin) markers was analyzed by immunofluorescence stains in SNU-449-CTNND1 cells and control cells. **h** Expression of epithelial and mesenchymal markers was analyzed by qRT-PCR in SNU-449-CTNND1 cells and control cells. **, *P* < 0.01 is based on the Student *t* test. All results are from three independent experiments. Error bars, SD

Next, we observed the morphological changes, and found that SK-Hep-1-shCTNND1 #1 cells reverted to an epithelial phenotype as compared to their respective control cells (Fig. 6a). Consistent with this, silencing of CTNND1 increased the levels of epithelial markers (E-cadherin and α-catenin), and decreased the levels of mesenchymal markers (N-cadherin and vimentin) (Fig. 6b-d). Conversely, SNU-449-CTNND1 cells exhibited a fibroblastic morphology (Fig. 6e). This observation was confirmed by expression analyses of epithelial and mesenchymal markers. We showed that CTNND1 overexpression decreased the levels of epithelial markers and increased the levels of mesenchymal markers (Fig. 6f-h). Taken together, these findings suggest that CTNND1 plays an important role in regulating EMT-MET plasticity of HCC cells.

### CTNND1 enhances Wnt/β-catenin signaling in HCC cells

**Fig. 7:**
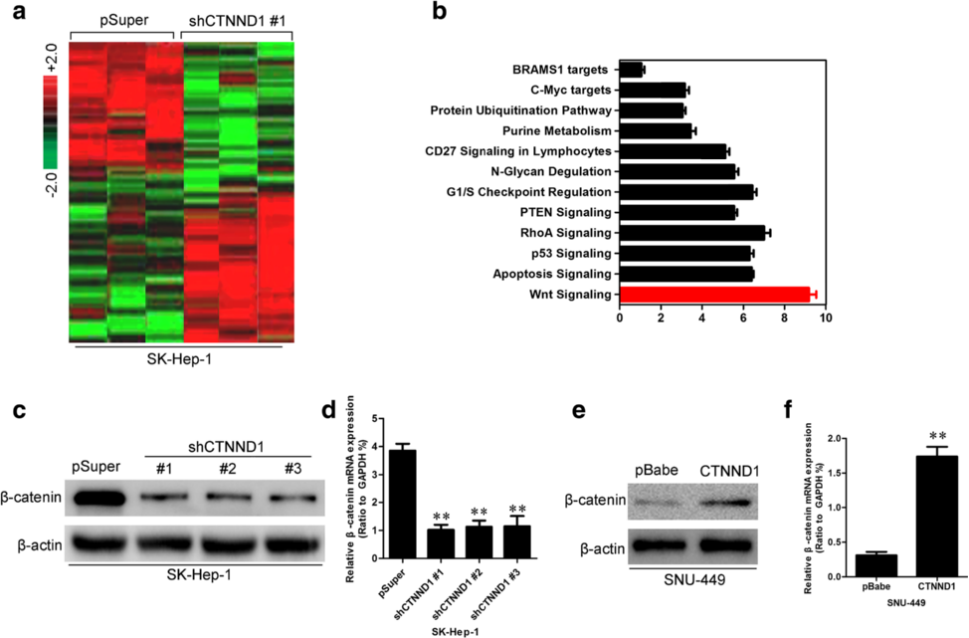
CTNND1 enhances Wnt/β-catenin signaling in HCC cells. **a** Supervised hierarchical clustering of genes differentially expressed after CTNND1 silencing in SK-Hep-1 cells. **b** Gene-set enrichment analysis was carried out using ConceptGen. **c** Expression of β-catenin protein was measured by Western blotting in SK-Hep-1-shCTNND1s cells and theirs control cells. **d** Expression of β-catenin mRNA was measured by qRT-PCR in SK-Hep-1-shCTNND1s cells and theirs control cells. **e** Expression of β-catenin protein was measured by Western blotting in SNU-449-CTNND1 cells and control cells. **f** Expression of β-catenin mRNA was measured by qRT-PCR in SNU-449-CTNND1 cells and control cells. **, *P* < 0.01 is based on the Student *t* test. All results are from three independent experiments. Error bars, SD

To better understand the mechanisms by which CTNND1 influences HCC development and progression, we performed gene expression profiling on the SK-Hep-1-shCTNND1 #1 cell line and its control cells. Microarray analyses identified a list of genes that were significantly differentially expressed after CTNND1 silencing, including downregulation of Wnt/β-catenin signaling (Fig. 7a). Furthermore, gene-set enrichment analysis indicated that gene signatures related to proliferation, neoplasm metastasis and invasion, cell movement and motility, and Wnt/β-catenin signaling were significantly changed in CTNND1-silenced cells (Fig. 7b), supporting the idea that CTNND1 regulates proliferation, EMT, and cancer invasion and metastasis.

These data also led us to hypothesize that CTNND1 exerts these functions via regulation of Wnt/β-catenin signaling. To test this, we first determined whether β-catenin is a downstream target of CTNND1 in HCC cells. Expression of β-catenin in the cells with altered CTNND1 expression was evaluated by qRT-PCR and Western blotting. Silencing CTNND1 in SK-Hep-1 cells dramatically decreased β-catenin mRNA and protein levels (Fig. 7c and d), whereas SNU-449-CTNND1 cells exhibited greatly increased β-catenin protein and mRNA levels (Fig. 7e and f).

**Fig. 8:**
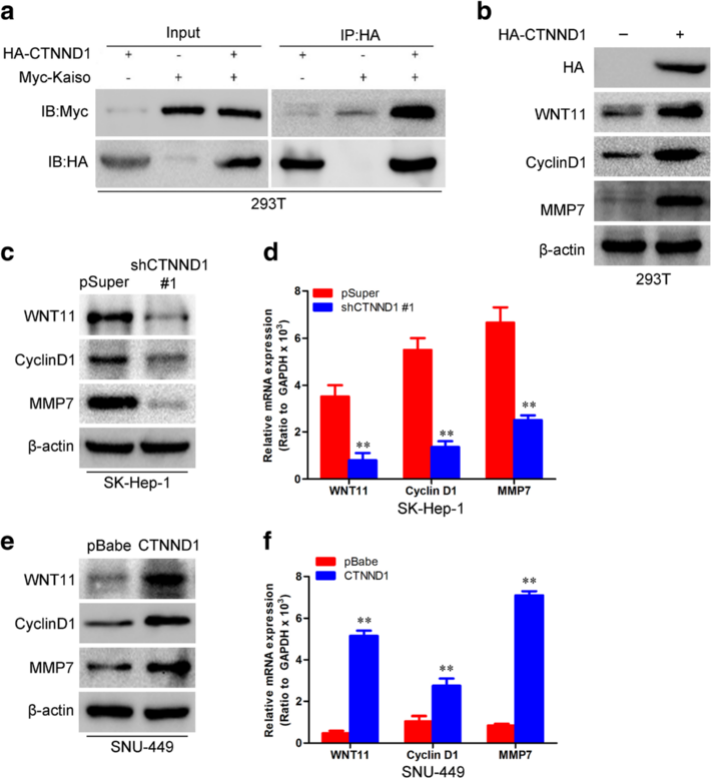
CTNND1 direct interacts with Kaiso and promotes WNT11, Cyclin D1, and MMP7 expression. **a** 293T cells were co-transfected with HA-tagged CTNND1 and Myc-tagged Kaiso constructs. Immunoprecipitation and subsequent immunoblot analysis revealed that HA-tagged CTNND1 co-immunoprecipitated with Myc-tagged Kaiso. **b** Expression of WNT11, Cyclin D1, and MMP7 protein was measured by Western blotting in 293T cells transfected with HA-tagged CTNND1 and control cells. **c** Expression of WNT11, Cyclin D1, and MMP7 protein was measured by Western blotting in SK-Hep-1-shCTNND1 #1 cells and control cells. **d** Expression of WNT11, Cyclin D1, and MMP7 mRNA was measured by qRT-PCR in SK-Hep-1-shCTNND1 #1 cells and control cells. **e** Expression of WNT11, Cyclin D1, and MMP7 protein was measured by Western blot in SNU-449-CTNND1 cells and control cells. **f** Expression of WNT11, Cyclin D1, and MMP7 mRNA was measured by qRT-PCR in SNU-449-CTNND1 cells and control cells. **, *P* < 0.01 is based on the Student *t* test. All results are from three independent experiments. Error bars, SD

It has been shown that CTNND1 regulates Wnt/β-catenin signaling through its direct interaction with the transcriptional repressor Kaiso in other cancers [23]. We next assayed whether CTNND1 directly interacts with Kaiso. As shown in Fig. 8a, 293T cells were co-transfected with HA-tagged CTNND1 and Myc-tagged Kaiso constructs. Immunoprecipitation and subsequent immunoblot analysis revealed that HA-tagged CTNND1 co-immunoprecipitated with Myc-tagged Kaiso, confirming a physical interaction between the two proteins. It has been shown that β-catenin can regulate WNT11, Cyclin D1, and MMP7 in many tumor cells [24], and so we next examined the role of CTNND1-mediated modulation of these genes. Of note, ectopic CTNND1 remarkably increased the level of WNT11, Cyclin D1, and MMP7 at both the protein and mRNA level (Fig. 8b, e, and f), whereas silencing CTNND1 in SK-Hep-1 cells robustly suppressed WNT11, Cyclin D1, and MMP7 expression (Fig. 8c and d).

**Fig. 9:**
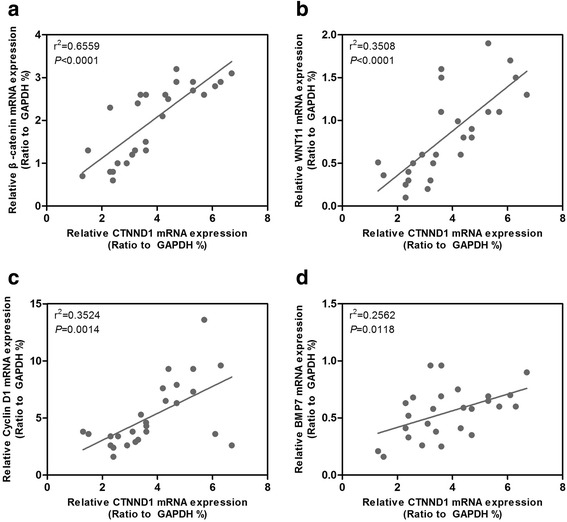
CTNND1 expression positively correlates with β-catenin, WNT11, Cyclin D1, and MMP7. **a** Correlation between CTNND1 expression and β-catenin expression in HCC biopsy samples was analyzed by correlation analysis. **b** Correlation between CTNND1 expression and WNT11 expression in HCC biopsy samples was analyzed by correlation analysis. **c** Correlation between CTNND1 expression and Cyclin D1 expression in HCC biopsy samples was analyzed by correlation analysis. **d** Correlation between CTNND1 expression and the MMP7 expression in HCC biopsy samples was analyzed by correlation analysis

To determine whether any correlation existed between CTNND1 expression and the representative markers of Wnt/β-catenin signaling in HCC biopsy samples, we obtained RNA from 26 HCC samples and analyzed CTNND1, β-catenin, WNT11, Cyclin D1, and MMP7 expression using real-time RT-PCR. We found that CTNND1 expression positively correlated with β-catenin, WNT11, Cyclin D1, and MMP7 (Fig. 9a–d). Taken together, these results show that CTNND1 mediates migration, invasion, and metastasis in HCC cells may partly by activation of Wnt/β-catenin signaling.

### Wnt/β-catenin signaling is a mediator for CTNND1-induced proliferative migration, and invasion in HCC cells

**Fig. 10:**
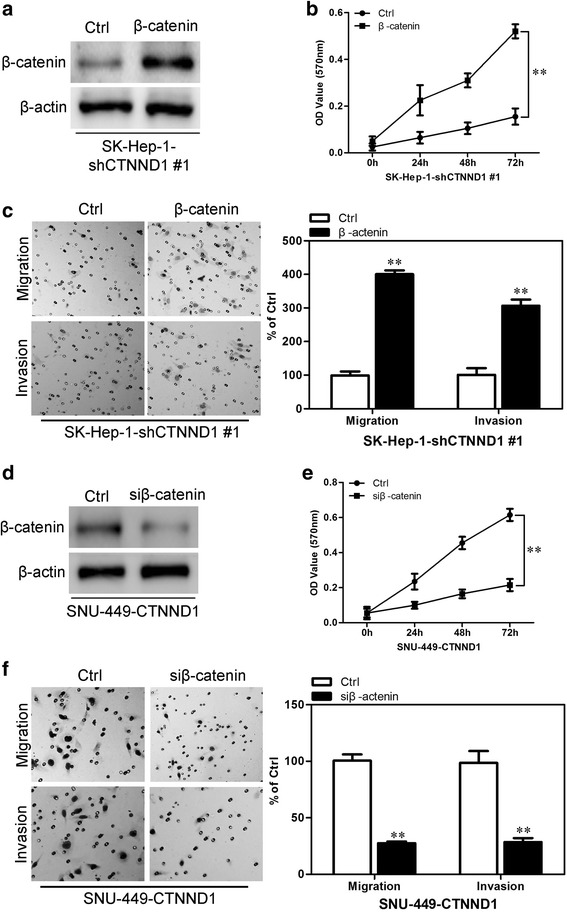
Wnt/β-catenin signaling is a mediator for CTNND1-induced proliferative migration, and invasion in HCC cells. **a** the expression of β-catenin was analyzed by Western blotting in ectopic expression β-catenin in SK-Hep-1-shCTNND1 #1 cells. **b** The proliferation of the SK-Hep-1-shCTNND1-β-catenin cell line and its control cells was examined by MTT assay. **c** indicated cells were subjected to Transwell migration (*top*), and Matrigel invasion assays (*bottom*), quantification of migrated cells through the membrane and invaded cells through Matrigel of each cell line are shown as proportions of their vector controls. **d** the expression of β-catenin was analyzed by Western blotting in β-catenin knocking down cells. **e** The proliferation of the SNU-449-CTNND1-siβ-catenin cell line and its control cells was examined by MTT assay. **f** indicated cells were subjected to Transwell migration (*top*), and Matrigel invasion assays (*bottom*), quantification of migrated cells through the membrane and invaded cells through Matrigel of each cell line are shown as proportions of their vector controls. ^**^, *P* < 0.01 is based on the Student *t* test. All results are from three independent experiments. Error bars, SD

On the basis of the indispensable role of Wnt/β-catenin signaling in the biologic functions of CTNND1, we overexpressed β-catenin in SK-Hep-1-shCTNND1 #1 cells (Fig. 10a) and silenced β-catenin in SNU-449-CTNND1 cells (Fig. 10d). Of note, β-catenin overexpression significantly increased proliferation in SK-Hep-1-shCTNND1 #1 cells (Fig. 10b). Moreover β-catenin overexpression significantly increased the migration, and invasion of SK-Hep-1-shCTNND1 #1 cells (Fig. 10c). Meanwhile silencing β-catenin decreased proliferation in SNU-449-CTNND1 cells (Fig. 10e). Moreover silencing β-catenin in SNU-449-CTNND1 cells significantly decreased the migration and invasion (Fig. 10f). Taken together, these results show that Wnt/β-catenin signaling mediates CTNND1-induced proliferation, migration and invasion in HCC cells.

## Discussion

In this research, we delineated for the first time the clinical significance of CTNND1 in HCC and the mechanistic role of CTNND1 in regulating HCC cell proliferation and metastasis. To our knowledge, this is the first study to show that CTNND1 plays a functional role in HCC cell proliferation, migration, invasion, and metastasis. CTNND1 overexpression in HCC cells induced EMT, migration, and invasion traits in vitro and enhanced metastatic capacity in vivo. In contrast, silencing CTNND1 reversed these events in otherwise aggressive and invasive HCC cells. We also showed that a mechanistic link exists between CTNND1 and Wnt/β-catenin signaling through direct interaction with the transcriptional repressor Kaiso, which subsequently leads to transcriptional upregulation of Wnt/β-catenin signaling expression. These results lead us to propose a model for CTNND1 regulation of EMT, migration, invasion, and metastasis through regulation of Wnt/β-catenin signaling in HCC cells.

The putative role of CTNND1 as an oncogene in cancer development is supported by the observations that CTNND1 is highly expressed in some tumors relative to normal tissues [25]. It is well recognized that inactivation of E-cadherin leads to cytosolic translocation of CTNND1 in colon, breast, bladder, lung, pancreas, prostate, and stomach tumors, which has been associated with tumor malignancy [13]. Indeed, studies in breast and colon cancer indicate that cytosolic CTNND1 controls the invasive phenotype of E-cadherin-deficient tumor cells [26]. Furthermore, a switch from E-cadherin expression to P-cadherin, which is associated with a more invasive behavior, in ovarian and pancreatic cancer cell lines results in the translocation of CTNND1 to the cytosol, which in turn induces cell migration by activating RAC1 and CDC42 [9]. Our study points to a novel function of CTNND1 in HCC metastasis through promoting two essential characteristics of metastatic disease in HCCs: migration and invasion [27, 28]. First, HCC cells expressing high levels of CTNND1 displayed an EMT phenotype, including the associated stimulatory effects on in-vitro migration and invasion. Interestingly, our results indicate that CTNND1 not only promotes EMT, but silencing of CTNND1 also leads to MET. Thus, CTNND1 promotes characteristics of migration that have been proposed as a model for cancer progression and metastasis. All of these characteristics that are induced by CTNND1 in vitro culminated in increased numbers of distant metastases in vivo. These empirical findings provide a mechanistic framework to explain the clinical observations that HCC patients with high levels of CTNND1 in tissue samples have more chance of distant metastasis, and a significantly shorter overall and disease-free survival.

The roles of several transcription factors as EMT regulators have been extensively reported [29–31]. In our effort to elucidate the mechanism by which CTNND1 modulates metastasis traits in HCC cells, we identified the Wnt/β-catenin signaling as an effective mediator of CTNND1-induced phenomena. It has been implicated that oxidative stress in the tumor microenvironment enhances canonical Wnt/beta-catenin signaling cascade, which is involved in the collective cell migration of the several kinds of solid tumors [32]. In this study, we found that modulation of CTNND1 expression altered the Wnt/β-catenin signaling. Previous studies have shown that CTNND1 can modulate gene expression through its direct interaction with the transcriptional repressor Kaiso [11]. Kaiso is a member of the BTB family of transcription factors that can bind to methylated DNA and/or a specific Kaiso binding sequence. Binding of CTNND1 to Kaiso might inhibit its DNA binding and relieve the repression of its target genes, such as WNT11, CyclinD1, and MMP7, which have been linked to non-canonical and canonical WNT-dependent developmental processes [13]. In accordance with previous studies, in the present study, we also found that ectopic CTNND1 remarkably increased the level of β-catenin, WNT11, Cyclin D1, and MMP7 at both the protein and mRNA level, whereas silencing CTNND1 robustly suppressed β-catenin, Wnt11, Cyclin D1, and MMP7 expression in HCC cells. Previous study has found that activation of canonical Wnt/β-catenin signaling enhances in vitro motility of cancer cells by activation of ZEB1 and other activators of epithelial-to-mesenchymal transition [33]. Thus, the CTNND1 induced activation of Wnt/β-catenin signaling promotes HCC cells ETM formation may partly by ZEB1.

Migration, invasion, and metastasis properties are essential for HCC cells to disseminate from adjacent tissues and to seed new tumors in distant sites. Our results demonstrate that CTNND1 regulated these essential characteristics of metastatic disease, and that CTNND1-induced processes are reversible with the suppression of CTNND1 expression, thus providing us with a potential therapeutic option to manipulate CTNND1 levels in clinical HCC practice.

## Additional file

Additional file 1: Figure S1. The expression of CTNND1 was analysised with TCGA data. (JPG 115 kb)
