# Supplementary figures and images for: Overexpression of CTNND1 in hepatocellular carcinoma promotes carcinous characters through activation of Wnt/β-catenin signaling
Source: J Exp Clin Cancer Res. 2016 May 18;35:82. doi: 10.1186/s13046-016-0344-9 (PMC4872337; doi:10.1186/s13046-016-0344-9)

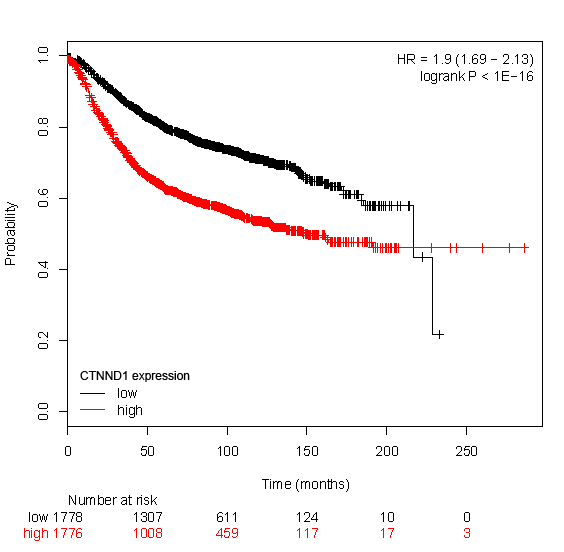

Supplement: Additional file 1: Figure S1. — The expression of CTNND1 was analysised with TCGA data. (JPG 115 kb) [file 13046_2016_344_MOESM1_ESM.jpg]
